# Efficiency of Expired Drugs Used as Corrosion Inhibitors: A Review

**DOI:** 10.3390/ma16165555

**Published:** 2023-08-10

**Authors:** Nicolae Vaszilcsin, Andrea Kellenberger, Mircea Laurentiu Dan, Delia Andrada Duca, Valentin Laurentiu Ordodi

**Affiliations:** Faculty of Industrial Chemistry and Environmental Engineering, Politehnica University Timisoara, Piata Victoriei No. 2, 300006 Timisoara, Romania; nicolae.vaszilcsin@upt.ro (N.V.); andrea.kellenberger@upt.ro (A.K.); duca.delia@gmail.com (D.A.D.); valentin.ordodi@upt.ro (V.L.O.)

**Keywords:** corrosion inhibitors, expired drug, inhibitory efficiencies

## Abstract

Corrosion inhibitors represent one of the most commonly used methods for significantly reducing the corrosion rate of metals and alloys. Adsorption inhibitors have a wide range of applications in cooling water systems, deicing solutions for aircrafts, airports and ways, etching and degreasing solutions, oil pipelines, paints and coatings and metal processing solutions. Adsorption corrosion inhibitors of metals and alloys are generally organic compounds that contain structures with heteroatoms (N, P, S, As, O) in their molecules, having lone pair electrons or π electrons in aromatic rings or multiple bonds. They enable relatively strong interactions between the metal atoms and organic molecules, resulting in a protective layer of organic molecules adsorbed at the metal–corrosive solution interface. Most molecules of active substances from drugs contain similar structures, which is why many drugs have been already tested as corrosion inhibitors. One of the major disadvantages of using drugs for this purpose is their particularly high price. To overcome this impediment, the possibility of using expired drugs as corrosion inhibitors has been investigated since 2009. The present paper is an exhaustive compilation of the scientific published papers devoted to the use of expired drugs as corrosion inhibitors in various aggressive solutions. The inhibitory efficiencies of expired drugs are presented as a function of the studied metal or alloy and the nature of the aggressive solution, as well as the concentration of the inhibitor in such a solution. Research has especially been focused on mild and carbon steel and less on stainless steel, as well as on some metals such as copper, zinc, nickel, tin and aluminum and its alloys. The experimental methods used to assess the inhibitory efficiencies of expired drugs are briefly discussed. Also, the available information on the stability of the active substances in the drugs is presented, although most authors were not concerned with this aspect. Finally, several actions are revealed that must be undertaken by researchers so that the results obtained in the study of the anticorrosive action of expired drugs can be applied at the industrial level and not remain only an academic concern.

## 1. Introduction

The damage caused by the corrosion of metals and metal alloys is considerable in many areas of human activity, such as industrial production, agriculture, housework, buildings and utilities, transportation and infrastructure. In these fields, new metallic materials are being continuously developed, with properties that more closely match the proposed objectives and that are obviously more affordable. According to the US National Association of Corrosion Engineers, the annual cost of corrosion is around USD 2.5 trillion [1]. These data justify further research on corrosion processes in order to identify new protection methods against this harmful phenomenon. There are several methods to protect metallic materials against corrosion; one of these concerns the use of corrosion inhibitors.

Corrosion inhibitors are chemical substances that, added to the aggressive environment, mitigate the metal degradation process. Inhibitors are typically added to the corrosive medium at very low concentrations—from tens of ppm to several thousands of ppm—in extremely aggressive conditions. The chemical nature of the inhibitors depends not only on the nature of the metallic material but also on the environment in which it is used, as well as on the working conditions, e.g., a thermal or hydrodynamic regime [2]. It is widely known that corrosion inhibitors are applied in a large variety of environments, including thermal agents, pickling and washing solutions, deicers, liquids for mechanical processing of metals, crude oils and liquid fuels, processing liquids from pharmaceutical and food industries, etc.

There are several classification criteria for inhibitors; the most important ones refer to the nature of the substances used or to the inhibited electrochemical reaction. According to the first classification criterion, inhibitors can be inorganic or organic compounds, while the second criterion seems more rigorous, dividing the inhibitors into three categories: anodic, cathodic and mixed inhibitors.

Anodic inhibitors act on the metal ionization process. They can be substances that form protective layers on the metal anodic sites or oxidants that can be reduced at a sufficiently high rate, causing the anodic current density to exceed the critical current density at which the metals reach the passive state. On the other hand, cathodic corrosion inhibitors diminish the hydrogen evolution reaction or oxygen reduction rate or form slightly soluble films on the metal cathodic sites, which limits the cathodic process coupled with metal ionization [3].

An important class of inhibitors is given by organic compounds that have the ability to adsorb on the metal surface, thus blocking the diffusion of participating chemical particles (reactants and reaction products) in the global corrosion process. Such compounds are known as adsorption inhibitors.

The most effective organic compounds as corrosion inhibitors are those that contain in their molecule heteroatoms lone pair electrons (N, P, S, As, O) or π electrons in aromatic rings or multiple bonds [4,5].

The legislative constraints imposed on the use of toxic and dangerous substances have focused the research of corrosion inhibitors towards environmentally friendly compounds. Thereby, the concept of *green corrosion inhibitors* has emerged. This category includes substances that are usually natural or similar to natural ones that meet the rules regarding toxicity, biodegradability and bioaccumulation [6,7]. Research in this field originally started with the study of natural plant extracts (roots, stem bark, flowers, fruits and seeds) and was then further stimulated by the abundance of natural resources. A series of substances with corrosion-inhibiting properties were identified in natural extracts, including polyphenols, alkaloids, tannins, flavonoids, ascorbic acid, sorbitol, methionine, maleic acid, glycosides, caffeine, proteins, amino acids, theophylline, coumarin and others [8,9], providing a consistent review on the use of plant extracts as green inhibitors for steel. The authors analyzed the anticorrosive effects of the extracts, presenting the most suitable solvents for the extraction of useful compounds, e.g., acetone for flavonols and tannins; chloroform for flavonoids and terpenoids; ethanol for alkaloids and polyphenols; ether for alkaloids, coumarins, fatty acids and terpenoids; methanol for anthocyanins, flavones, lactones, polyphenols and terpenoids; water for anthocyanins, polypeptides, tannins, terpenoids and starch. The state of the art in the field of green inhibitors for steel was pertinently analyzed by [10], who identified four types of bonds between the organic substrate and the iron surface: electrostatic interactions between the electric charge of the inhibitor molecule and the opposite charge of the metal (physical adsorption); interactions between protonated inhibitors and the excess of negative charges of the metal (usually, in practical applications, both physical and chemical adsorption take place, especially in an acidic environment (predominantly physical adsorption)); interactions of a chemical nature between the π or lone pair electrons of the organic substance and the 3*d* orbitals of iron atoms (chemical adsorption); the formation of protective chelate films (chemical reaction).

Looking for other green inhibitors, the interest of researchers has focused on the active substances from drugs because the vast majority of them contain some molecular structures that favor the adsorption process at the metal–aqueous solution interface. Among the first drugs studied as corrosion inhibitors were antibiotics such as Ampicillin, Cloxacillin, Flucloxacillin and Amoxicillin, whose inhibitory efficiencies reached up to 90% [11]. In the same solutions, antihypertensive drugs (Enalapril maleate, Atenolol and Etilefrine) were successfully tested [12]. Cellulose acetate films doped with Amoxicillin were also shown to be effective for the anticorrosive protection of aluminum in sodium chloride solutions [13]. Arslan et al. [14] reported an inhibition efficiency of up to 95% for carbon steel in acid solutions, using sulfamide drugs such as Sulfaguanidine, Sulfamethazine, Sulfametaxazole and Sulfadiazine. Sudhish et al. [15] managed to inhibit with high effectiveness (88.5%) the corrosion of carbon steel in 1 M hydrochloric acid solution using Streptomycin (a commercially available drug in pure state). Cefazolin, Ceftazidin and Cefatrexil have shown good inhibitory efficiency for carbon steel in acidic solutions [16,17,18]; also for carbon steel in acid environments, inhibitory efficiencies of up to 92% have been reported for Trimethoprim [19]. A useful inhibitor for reducing the corrosion rate of copper in potassium nitrate solution is Caffeine (inhibitory efficiency up to 66%) [20] and Chitosan is suitable for acidic solutions (inhibitory efficiency up to 95%) [21]. As expected, some antibiotics reduce the corrosion rate of metals produced in the presence of sulfate-reducing bacteria [22]. In an excellent review, Gece [23] identified 17 classes of drugs that can be used as corrosion inhibitors for various metals and alloys (carbon and stainless steel, Al and alloys of Al, Cu, Zn and Ti), in various corrosive environments (HCl solutions, H_2_SO_4_, H_3_PO_4_ and NaCl). The 17 drug classes refer to b-lactam antibiotics, quinolones, tetracyclines, macrolides, lincosamides, sulfonamides, aminoglycosides, antifungal drugs, anthelmintic drugs, muscle relaxants, antiviral drugs, opioid analgesics, histamines, antipsychotic drugs and antihypertensive drugs. The article published by Gece had a great impact on researchers in the field of corrosion protection, driving the studies on drugs as corrosion inhibitors. On the other hand, there were also arguments against the use of corrosion-inhibiting antibiotics regarding the risk of reaching the environment, which could accelerate the induction of antibiotic resistance to some dangerous microorganisms [24].

The study of drug inhibitory properties has advanced remarkably in recent years. A number of other drugs have been identified with significant anticorrosive inhibitory efficiency for a large number of metallic materials in various aggressive solutions. Recently, Verma et al. [25] published a review based on 198 references analyzing the existing potential for using drugs as corrosion inhibitors. The authors highlighted the benefits of this type of anticorrosive protection, but also a number of drawbacks that limit large-scale practical applications, such as high costs of active substances, high toxicity of some drugs that can reach the environment, negative influences on organisms and low solubility in certain aggressive media.

In an effort to avoid the high costs of drugs used as corrosion inhibitors, corrosion researchers explored the use of expired drugs [26]. The first mention in this regard was made by R. A. Hameed [27], who used expired Ranitidine as a corrosion inhibitor for aluminum in 1M hydrochloric acid. In addition to expired drugs, unused drugs due to patient refusal, as well as drugs that do not meet the pharmaceutical quality standards, can also be considered. It was revealed that the use of expired drugs can solve two more sensitive problems, i.e., limiting environmental pollution with drugs and reducing drug disposal costs [28].

The aim of the paper is to present the results obtained in the last decade in the research of expired or unused drugs as corrosion inhibitors for metals and alloys frequently used in industry. Gece [23], Chauhan et al. [29] and Baari et al. [30] have presented inhibitory efficiencies depending on the drug class. This classification is irrelevant for practical applications since, at the industrial level, the protection of certain metals or alloys is requested as a function of the nature of the aggressive environment. In a recent review, Verma et al. analyzed emerging trends in using pharmaceutically active compounds as corrosion inhibitors in aqueous solution depending on the nature of the corrosive environment [25]. In this work, a different classification was used, first of all, by taking into account the nature of the metal and then by the corrosive environment. In this way, opportunities of the practical application of expired drugs as corrosion inhibitors are directly highlighted.

## 2. Inhibitory Efficiency of Expired Drugs

### 2.1. Experimental Methods

One of the most important parameters characterizing the ability of an organic compound to limit the corrosion rate of metals or alloys in an aggressive environment is the inhibitory efficiency (*IE*) (defined by Equation (1)), calculated by the weight loss method [31].

(1) $$IE=\frac{K_{o}-K_{inh}}{K_{o}}\cdot 100\%$$

where *K_o_* is the corrosion rate in the absence of the inhibitor, mg m^−2^ h^−1^; *K_inh_* is the corrosion rate in the presence of the inhibitor, mg m^−2^ h^−1^.

The inhibition efficiency can also be calculated based on the corrosion current densities obtained by potentiodynamic polarization or electrochemical frequency modulation, using Equation (2) [32].

(2) $$IE=\frac{i_{corr\left(o\right)}-i_{corr\left(inh\right)}}{i_{corr\left(o\right)}}\cdot 100\%$$

where *i_corr_*_(_*_o_*_)_ is the corrosion current density in the absence of the inhibitor, A m^−2^; *i_corr_*_(_*_inh_*_)_ is the corrosion current density in the presence of the inhibitor, A m^−2^.

Also, the inhibitory efficiency can be calculated based on the charge transfer resistances, *R_ct_*, available through electrochemical impedance spectroscopy, according to Equation (3) [33].

(3) $$IE=\frac{R_{ct\left(inh\right)}-R_{ct\left(o\right)}}{R_{ct\left(inh\right)}}\cdot 100\%\;$$

where *R_ct*(*o*)*_* and *R_ct*(*inh*)*_* stand for the charge transfer resistance in the absence and presence of the inhibitor, respectively.

There are several methods to evaluate the corrosion rate; the most frequently employed are as follows: weight loss measurement, volumetric method, potentiodynamic polarization, electrochemical impedance spectroscopy, electrochemical frequency modulation and thermometric measurements. All these methods have also been applied in research related to the testing of expired drugs as corrosion inhibitors.

*Weight loss measurement* (WL) is the most relevant method to estimate the corrosion rate, based on the weight of the metal sample before and after immersion in the corrosive environment. To obtain accurate results, the corrosion has to be as uniform as possible; moreover, the corrosion products need to be completely removed from the metal surface without affecting its integrity. The main disadvantage of this method is the long duration of the tests, sometimes lasting several months. It should be specified that the gravimetric method gives an average value of the corrosion rate during the duration of the test. It is generally accepted that an inhibitor is suitable for the corrosive environment if the *IE* is higher than 90%, preferably close to 100%, at concentrations of the order of tens or hundreds of ppm, at which the corrosion rate does not exceed 0.1 mm/year [34]. Of course, this limit is considerably lower in circumstances where the metal ions resulting from the corrosion process can contaminate the products, e.g., in the food or pharmaceutical industry.

*Volumetric method* (VOL) is less used as it is only applied in situations where the cathodic process coupled with the ionization of the metal is exclusively hydrogen evolution. Like the gravimetric method, the volumetric method gives an average value of the corrosion rate related to the duration of the experiment. It generally applies to pure metals [35].

*Potentiodynamic polarization* (PDP) is a much more expeditious method and, consequently, it is preferred by most researchers. If the electrode potential is shifted too far in the anodic direction, this can cause advanced corrosion of the sample, transforming this procedure into a destructive testing method. This method involves recording the linear voltammograms *i* = f(*E*), where *i* is the current density (A m^−2^) and *E* is the electrode potential (V). The scan rate or potential sweep rate in potentiodynamic corrosion measurements recommended by ASTM standards is 0.6 V h^−1^, which corresponds to 0.166 mV s^−1^. However, higher scan rates, up to 1 mV s^−1^, are usually applied in PDP corrosion measurements, considering they are still low enough to assure quasi-potentiostatic conditions. Based on this dependence, diagrams *E* = f(*lg i*) are obtained, from which the corrosion current density *i_corr_* and the corrosion potential *E_corr_* are then calculated by the Tafel extrapolation method. Finally, inhibitory efficiency (*IE*) is obtained based on Equation (2). The values of the corrosion current densities obtained by the PDP method are quasi-instantaneous and, therefore, may differ significantly from the values obtained by the WL method [36,37]. Another important parameter obtained by the PDP method is the corrosion potential, which gives information about the inhibitory mechanism. The shift of the corrosion potential to more positive values shows that the inhibitor is anodic, whereas the shift to more negative values means that the inhibitor is cathodic.

*Electrochemical impedance spectroscopy* (EIS) is a non-stationary technique that consists of applying a low-amplitude alternating signal (potential) to the working electrode, polarized at a constant potential. For corrosion tests, the potential at which the metal sample is polarized is the open circuit potential, therefore this technique is a non-destructive method for corrosion resistance assessment. Impedance spectra provide a series of information, of which relevant for metal corrosion is the charge transfer resistance *R_ct_*. Knowing the values of this parameter, in the presence and absence of the corrosion inhibitor, the inhibitory efficiency can be calculated based on Equation (3) [38]. The EIS allows the evaluation of the double layer capacitance that can be used in corrosion mechanism research. As in the case of the PDP slope method, the inhibitory efficiencies obtained by electrochemical impedance spectroscopy are of quasi-instantaneous values. As a rule, these values are used to validate the results obtained by the PDP method.

*Atomic absorption spectroscopy* (AAS) is similar to the gravimetric method, with the difference that the mass of dissolved metal is determined based on the content of metal ions in the aggressive solution. It is applied especially in the case of carbon steel, where the content of Fe^2+^ ions in the test solution is determined by AAS [39], but also in the case of pure metals [40].

*Electrochemical frequency modulation* (EFM) is also a non-stationary method applied in the evaluation of corrosion currents, based on the simultaneous application of two sinusoidal potentials on the metal–solution interface. Values obtained using EFM are represented as currents as a function of frequency, known as intermodulation spectrum. Analysis of the metal–aggressive solution interface response provides information that allows obtaining the corrosion current densities [41,42].

*Thermometric measurements method* (TM) consists of the evaluation of the heat evolved during the corrosion tests. The temperature in the metal sample–aggressive solution is monitored in time, starting with the initial temperature *T_i_* up to the maximum temperature value *T_m_*. The reaction number *RN* is defined by Equation (4) [43].

(4) $$RN=\frac{T_{m}-T_{i}}{t}$$

where *t* is the time to attain the maximum temperature.

Inhibitory efficiency is calculated using Equation (5).

(5) $$IE=\frac{RN_{o}-RN_{inh}}{RN_{o}}\cdot 100\%$$

where *RN_o_* and *RN_inh_* are the reaction numbers in the absence and presence of the inhibitor, respectively.

*Acidimetric analysis* (ACI) is a method that is seldom applied. It consists of the evaluation of H_3_O^+^ concentration in the corrosive solution before and after dipping the metal both with and without inhibitor. Inhibitory efficiency is given by Equation (6).

(6) $$IE=\left(1-\frac{\Delta H_{inh}^{+}}{\Delta H_{o}^{+}}\right)\cdot 100\%$$

where $\Delta H_{inh}^{+}$ is the change of H_3_O^+^ concentration in a test solution with inhibitor; $\Delta H_{o}^{+}$ is change of H_3_O^+^ without inhibitor [44].

### 2.2. Anticorrosive Effect of Expired Drugs

Even though most of the studies on expired drugs as corrosion inhibitors refer to carbon steel, special attention has been paid to steel with low carbon content (maximum 0.3% carbon), commonly known as mild steel. The studies were carried out in aggressive environments (generally diluted solutions of hydrochloric acid and sulfuric acid), taking into account the practical possibilities of applications at an industrial level. Less addressed is stainless steel, considering the fact that, in general, in the test solutions it is corrosion resistant. There are several studies on the corrosion inhibition of copper and copper alloys, as well as aluminum and aluminum alloys. A few references also mention studies on nickel and tin. The inhibitory efficiencies for mild steel are shown in Table 1. For each case, the aggressive solution and concentration of the inhibitor are specified, as well as the method used to determine the inhibitory efficiency.

In highly aggressive solutions, such as dilute solutions of hydrochloric or sulfuric acid, a large number of expired drugs showed a good *IE* for mild steel, which in most cases exceeded 90%. Thus, Dahiya et al. [65] reported for expired Ethambutol *IE*s of up to 99%, determined by WL in 0.5 M HCl solution at 30 °C and a concentration of 1000 ppm. It was remarkable that this expired drug was active even at lower concentrations; for example, at 200 ppm Ethambutol, the *IE* was maintained at almost 90%. Also in hydrochloric acid solution, but at a concentration of 1 M, expired Atorvastatin proved to be a superior inhibitory *IE*: 88.2%, 92.3% and 99.1% (values determined by PDP), at relatively low concentrations: 50 and 100, respectively, 150 ppm. *IE*s obtained by EIS were 94.4%, 94.7% and 96.4%, while *IE* values of 88.8%, 94.1% and 96.5% were obtained by WL measurements at the same inhibitor concentrations. It has to be mentioned that the authors performed a comparison between the inhibitory effects of expired Atorvastatin and fresh Atorvastatin, reaching the conclusion that they were similar [49].

Raghavendra et al. [68] studied a series of expired drugs as mild steel inhibitors in 3M HCl solutions. Very good *IE*s were obtained for Abacavir at concentrations of 0.4 g L^−1^: 95% (WL), 94% (AAS) and 95% (PDP). In order to simulate the aggressiveness of the pickling solutions for mild steel, expired drugs were tested in the same solution but at a temperature of 60 °C. For expired Lorazepam (0.4 g L^−1^), the *IE*s were 82.7% (WL), 96.5% (AAS) and 87.7% (PDP) [86]; for expired Doxercalciferol (0.4 g L^−1^) were 95.4% (AAS), 87.0% (PDP) and 88.6% (EIS) [70]; for expired Ceftin (0.4 g L^−1^) were 93.0% (WL) and 94.4% (AAS) [64]; for expired Fluoxymesterone (0.2 g L^−1^) were 94.2% (AAS), 87.5% (PDP) and 88.8% (EIS) [71].

Dohare et al. [58] carried out studies regarding the values of the inhibitor concentration preferred in industrial applications, e.g., 50–100 ppm. In 1 M HCl solutions, for mild steel at 100 ppm expired Lumerax, the *IE*s were 98.5% (WL), 97.6% (EIS) and 95.2% (PDP); for expired Tramadol at the same concentration, the following *IE* values were obtained: 96.1 (WL), 97.2% (EIS) and 97.1% (PDP) [63]. Another potentially applicable expired drug (Cefdinir) was identified by Singh et al. [50]. It was active for mild steel in 1 M HCl solution at relatively low concentrations (200 ppm), at which inhibitory *IE*s had high values of 96.9% (EIS) and 95.6% (PDP). In the same conditions for expired Atenolol and Nifidipine, Gupta et al. [48] reported *IE*s of 91.0% (PDP) and 93.3 (EIS), respectively, 93.2% (PDP) and 95.6% (EIS). For expired Ranitidine, but at 400 ppm, Hameed [62] obtained *IE* values of 89% (WL), 90% (PDP) and 92% (EIS).

It is expected that when the concentration of HCl increases the amount of inhibitor required will be higher. In 2 M HCl solution, Chaudahari and Patel used 400 ppm expired Bactrim at a temperature of 30 °C, obtaining *IE*s of 67.9% (WL), 94.4% (PDP) and 93.1% (EIS); at a concentration of 500 ppm of expired Acetazolamide, *IE*s were 82.4% (WL), 93.3% (PDP) and 96.4% (EIS) [66].

Since, for mild steel, H_2_SO_4_ dilute solutions have a high aggressiveness, there are several studies dedicated to the identification of effective expired drugs to prevent the corrosive effect of this environment. The recent research of Abdallah et al. [73] on two expired antibiotics—Glibenclamide and Glimepiride—for the corrosive protection of mild steel in 0.5 M sulfuric acid has been published. At a temperature of 25 °C and an inhibitor concentration of 500 ppm, remarkable values of *IE* were obtained: 86.3% (WL), 85.1% (PDP) and 86.5% (EIS) for expired Glibenclamide, respectively; 86.3% (WL), 88.0% (PDP) and 90.4% (EIS) in the case of expired Glimepiride. In the same aggressive solution, 500 ppm expired Tramadol had good *IE*s of 93.2% (WL), 93.6% (VOL) and 94.0% (ACI) [44].

In 1 M H_2_SO_4_ solution, Ma et al. [78] obtained promising *IE* values using expired Formoterol at concentrations of 200 and 300 ppm at 50 °C (practiced in carbon steel pickling solutions). Using concentrations of 200 ppm expired Formoterol, the following values of *IE* were obtained: 91% (WL), 94% (EIS) and 90% (PDP), as well as 95% (WL), 98% (EIS) and 95% (PDP) at concentrations of 300 ppm. Moreover, in 1 M H_2_SO_4_ solution at 20 °C, Alfakeer et al. [75] tested the anticorrosive effects of expired drugs Ampicillin and Flucloxacillin. At an inhibitor concentration of 400 ppm, high *IE* values were obtained: 93% (WL), 94% (PDP) and 91% (EIS) for expired Ampicillin and 88% (WL), 91% (PDP) and 89% (EIS) for expired Flucloxacillin. Similar results have been obtained using expired Metoclopramide, Chlorpromazine and Mebeverine [51].

In solutions simulating sea water (3.5% NaCl), the *IE*s of expired Rabeprazole, Domperidone and Benfotiamine were reported as 98.5%, 98.8% and 98.9% (values obtained by WL). It is interesting that the *IE* values obtained by PDP were much lower (63%, 67% and 58%). This significant difference was explained by the fact that the values obtained by PDP were quasi-instantaneous and referred to the beginning of the corrosion process when the metal surface was clean. The WL values were obtained over a long period of time (5 days), when corrosion products accumulated on the metal surface and possible iron complexes with the inhibitors in the solution, which had a protective effect [81]. In a solution of 0.1 M Na_2_SO_4_ + 3% NaCl, Bustos-Terrones et al. [80] reported an *IE* of 84% for expired Fluconazole and 91% for expired Lanzoprazole. In both cases, the concentration of the inhibitor was 150 ppm.

A large number of references report research on carbon steel, having up to 2.1% carbon (Table 2). Due to its qualities (primarily its high hardness), it is the most used metallic material in industry, agriculture and construction. As in the case of mild steel, research on the corrosion inhibition of carbon steel has been particularly oriented towards aggressive dilute hydrochloric acid solutions.

Significant results were obtained by Fouda et al. [96] using expired Carvedilol as inhibitor in 1 M HCl solution at 25 °C and a concentration of 1.6 × 10^−4^ M, which is equivalent to approximately 65 ppm. The values obtained for *IE*s were as follows: 98.9% (WL), 98.1% (PDP), 98.6% (EIS) and 98.1% (EFM). In the same solution at 25–30 °C, expired Pantoprazole sodium showed an *IE* of over 90% at a concentration of 300 ppm [107]. More modest results were obtained with expired Clopidogrel (*IE* up to 86%) at 25 °C in 2 M HCl with a drug concentration of 250 ppm [112]. Very good results were obtained by Dohare et al. in 1 M HCl solution, even at slightly higher temperatures (35 °C), with reasonable concentrations of expired Podocip (100 ppm): 96.6% (WL), 97.9% (EIS) and 97.6% (PDP) [110]. Accurate results were published by Hameed [101] for expired Megavit. In 1 M HCl solution with 300 ppm inhibitor at 30 °C, the *IE* values determined by EIS and PDP exceeded 90%; at lower concentrations (50 ppm), the *IE* remained at high values (over 82%), which recommended the practical use of expired Megavit in solutions used in pickling operations. Similar results have been obtained by Motawea, using expired Nitazoxamide (*IE* over 90% in 1 M HCl with 300 ppm inhibitor at 25 °C) [103]. In the same conditions, considerable results have been obtained with expired Aripiprazole: 93.1% (WL), 94.8% (PDP) and 95.1% (EIS) [91]. Recently, better *IE*s have been reported for expired Augmentine: 97.8% (WL), 97.1% (PDP) and 98.0% (ACI) [92]; for expired Immulant: 97.5% (PDP) and 95.3% (EIS) [99]; as well as for expired Omeprazole: 96.6% (WL), 98.7% (PDP) and 98.0% (EIS) [105].

Duca et al. [108] compared the *IE*s of expired Paracetamol for carbon steel in 1 M HCl and 0.5 M H_2_SO_4_ solutions at the same drug concentrations (150 ppm). Using the rapid evaluation methods PDP and EIS, in the first solution, *IE*s of about 85% were obtained, and for the second solution, over 93%. The anticorrosive effect of expired Paracetamol was also confirmed in 1 M H_2_SO_4_ solution for mild steel by Al-Gorair and M. Abdallah [117]. They obtained *IE*s of 94.2% (PDP) and 92% (EIS) at concentrations of 275 ppm. Expired Paracetamol was also active in 2 M NaCl solution, where the addition of Zn^2+^ ions showed a synergistic effect. At 25 °C and a concentration of 200 ppm expired Paracetamol and 100 ppm Zn^2+^, the *IE* values were 91.0% (PDP), 92.1% (EIS), 92.0% (TM), 94.4% (WL) and 83.8% (AAS) [100]. As well, in diluted NaCl solution, for expired Zosyn (3.5% NaCl), *IE*s up to 91% were obtained [122]; in 3% NaCl, for expired Perindopril and Alprazolam, *IE*s reached values of 91.5% and 85.8%, respectively [121].

Prospective results have been reported for the anticorrosive properties of expired Esomeprazole for carbon steel in 1 M H_3_PO_4_. At an inhibitor concentration of 10^−4^ M, the following *IE*s were obtained: 99.55% (PDP) and 99.52% (EIS) [119]. In a more concentrated solution (3 M H_3_PO_4_), good *IE*s were obtained by Tajabadipour et al. using expired Lansoprazole and Rabeprazole [120].

In papers related to the anticorrosive protection of mild steel and carbon steel, researchers usually specified the elemental composition of their test samples, which differed within quite wide limits. In these circumstances, it could be stated that the authors’ results were not comparable, even if they were obtained under the same conditions. To avoid this impediment, M. Abdallah et al. used in their studies pure iron supplied by SABIC (Saudi Basic Industries Corporation), in which the iron concentration was generally 99.99% (Table 3). In this way, the influence of the complementary steel elements on the corrosion rate and implicitly on the *IE*s was largely removed. In 1 M HCl solution, expired drugs Ampicillin and Ceftriaxone were tested, at concentrations of no more than 500 ppm, for which, at 25 °C, *IE* values of 95.8% (PDP), 91.8% (WL) and 95.8% (EIS), respectively, 98.0% (PDP), 96.9% (WL) and 97.9% (EIS) were reported. But, even at 100 ppm concentrations of the inhibitors, *IE*s were maintained at values above 84%, respectively, 88%, which recommended the practical applications of these expired drugs [127]. Under the same conditions, values over 90% were obtained using expired Acyclovir and Omeprazole [128], as well as expired Amoxicillin and Cefuroxime [129].

In 0.5 M H_2_SO_4_ solution for SABIC iron, anticorrosive effects had expired Thiamine (B1 vitamin) and Riboflavin (B2 vitamin), which are truly “green inhibitors”. At concentrations of 250 mg L^−1^, *IE*s were located around 90% [130].

Hameed et al. conducted the only study on the anticorrosive protection of cast iron in 1 M HCl using expired Cefpodoxime, Levofloxacin, Ofloxacin and Linezolid; their results are summarized in Table 4 [131].

A study specifically dedicated to the anticorrosion protection of an X25 carbon steel pipeline was carried out by Eid [97]. The author tested expired Desloratidine as an inhibitor at a concentration of 1.93 × 10^−4^ M (approximately 60 ppm) in 1 M HCl at 30 °C and obtained *IE*s of 92.7% (WL) and 85.2% (PDP). For an X60 carbon steel pipeline in 15% HCl solution at 30 °C, relevant *IE*s were obtained: 91.5% (PDP), 92.4% (EIS) and 96.5% (WL), with expired Pregabalin but at relatively high concentrations of the inhibitor (1.5 g L^−1^) [132]. For carbon steel used for petroleum pipelines, tests with expired Co-amoxiclav (300 ppm) in 1 M H_2_SO_4_ at 30 °C revealed an *IE* of over 95% [133]. The results related to the inhibition efficiency of various drugs on the corrosion of pipeline steel are compared in Table 5 for different aggressive media.

The interest of researchers to identify expired drugs with anticorrosive properties for stainless steel was extremely low considering its high chemical resistance in aggressive environments. For stainless steel AISI 304L, an excellent anticorrosive action was recorded for expired Levothyroxine at concentrations of 150 ppm in 0.5 M H_2_SO_4_ solution at 25 °C: 94.3% (WL), 93.2% (PDP) and 91.7% (EIS) [135]. More modest results were obtained for AISI 304 using expired Condor in 2 M HCl [136] and Metronidazole in 1 M HCl [102]. Table 6 provides information on the inhibitory efficiency of some expired drugs relevant for corrosion protection of AISI 304 stainless steel.

Promising results have been obtained for copper in HCl solutions and especially in HNO_3_ (very aggressive for this metal) (Table 7). In 1 M HCl solution, expired Ceftazidime had *IE*s of 94.2% (WL), 93.1% (PDP) and 74.1 (EIS) at 25 °C and a concentration of 300 ppm [138]. At higher concentrations of HCl (5 M), contradictory results were obtained. Using expired Ixabepilone at an unusually low concentration (0.4 mg L^−1^), the following *IE* values were obtained: 97.6% (PDP), 76.4% (WL) and 86.2% (EIS), while with Neftifine (at a concentration 10 times higher), the *IE* values were 68% (WL), 97.2% (PDP) and 73.5% (EIS) [139]. An interesting study was carried out by Tasić et al. regarding the anticorrosive action of expired Ibuprofen for metallic copper in acid rain [140]. At relatively high concentrations of expired Ibuprofen (10^−2^ M), the *IE* values were 97.2% (PDP), 97.3% (EIS) and 96.5 (WL). The *IE*s were maintained at high values and at practically applicable concentrations of 10^−3^ M, for which the *IE*s were 91.6% (PDP), 96.0 (EIS) and 90.9 (WL). In the same acid rain, Paracetamol (10^−2^ M) showed *IE*s of 96.3% (PDP) and 97.2% (EIS) [141].

For α-Brass alloy (70% Cu, 30% Zn) in 1 M HCl solution at 25 °C at a concentration of 300 ppm, expired Ranitidine showed an *IE* of over 90% [142].

Although copper is a noble metal, nitric acid solutions are particularly aggressive due to the oxidizing properties of nitrate ions. For this reason, Fouda and Badawy tried to identify expired drugs with an inhibitory effect for copper in 1 M HNO_3_ [143]. The results for *IE*s obtained with expired Simvastatin at 300 ppm and at 25–30 °C were modest: 70.1% (PDP), 66.4% (EIS), 72.7% (EFM) and 68.1% (WL). On the contrary, under the same conditions with Meropenem, the results were remarkable: 94.6% (WL), 93.7% (PDP), 96.7% (EIS) and 98.7% (EFM) [142]. In 2 M HNO_3_ solution at 25 °C and a concentration of 400 of expired Megavit, the *IE*s did not exceed 67% [143]. In a more diluted solution (0.1 M HNO_3_), *IE* values of 92.9% (WL), 92.9% (PDP) and 90.1% (EIS) were obtained using 10^−4^ M Midazolam [144].

**Table 7:** Inhibitory efficiency of expired drugs for Cu corrosion.

Corrosive Solution	Expired Drug	Inhibitor Conc.	IE (%) (Experimental Method)	Reference
1 M HCl	Ceftazidime	300 ppm	94.2 (WL); 93.1 (PDP); 74.1 (EIS)	[138]
1 M HCl	Metronizadole	1 mmol L^−1^	91.8 (PDP)	[145]
1 M HCl	Ranitidine	300; 400; 300 ppm	92.4 (WL); 90 (PDP); 91.8 (EIS)	[142]
5 M HCl	Ixabepilone	0.4 mg/L	76.377 (WL); 97.599 (PDP); 86.246 (EIS)	[139]
5 M HCl	Naftifine	4 mg/L	68.511 (WL); 97.189 (PDP); 73.452 (EIS)	[88]
0.1 M HNO_3_	Midazolam	10^−4^ M	92.9 (WL); 92.9 (PDP); 90.1 (EIS)	[144]
1 M HNO_3_	Meropenem	300 ppm	94.6 (WL); 93.7 (PDP); 96.7 (EIS); 98.7 (EFM)	[146]
1 M HNO_3_	Simvastatin	300 ppm	78.8 (WL); 70.1 (PDP); 66.4 (EIS); 72.7 (EFM)	[143]
2 M HNO_3_	Megavit Zinc	400 ppm	66.87 (WL); 61.18 (PDP); 60.49 (EIS)	[147]
2 M HNO_3_	Tylosin	300 ppm	92.1 (WL); 94.1 (PDP); 93.7 (EIS)	[148]
Synthetic acid rain	Ibuprofen	10^−2^ M	96.5 (WL); 97.2 (PDP); 97.3 (EIS)	[140]
	Paracetamol	10^−2^ M	96.3 (PDP); 97.2 (EIS)	[141]

Aluminum—the second metal in terms of production and use worldwide— is more susceptible to corrosion both in acidic and basic environments due to the fact that it is much more electropositive than other common metals. Aluminum’s naturally occurring oxide layer (which forms when the metal comes into contact with the atmosphere) is porous and unable to protect the base metal against corrosion. For this reason, before certain operations such as anodizing, passivation or coloring, aluminum is subjected to the pickling process. Avoiding aluminum losses during contact with the pickling solution is achieved by adding corrosion inhibitors to the solution. Numerous expired drugs have been tested as inhibitors (Table 8). In 3 M HCl solution, which simulates pickling solutions, Raghavendra et al. successfully tested several expired drugs. With expired Atenolol at concentrations of 0.4 g L^−1^ and at temperatures practiced in pickling baths, *IE*s of 97.2% (WL), 97.0 (AAS), 92.1% (VOL) and 98.7 (PDP) were obtained [149]; with expired Oseltamivir (2 mg L^−1^): 91.3% (WL), 98.8% (AAS) and 98.6% (PDP) [150]; with expired Naproxen (1 g L^−1^): 99.0 (WL), 97.7% (EIS) and 97.2% (VOL) [151]. In more dilute solutions (1 M HCl) using expired Voltaren at a concentration of 125 ppm and at a temperature of 30 °C, the *IE* had values of approximately 90% [152]; with expired Cidamex (300 ppm and 25 °C) the *IE*s were between 81% and 91% [153]; with expired Ranitidine (300 ppm and 30 °C), the *IE* values were lower (82%) [27]. An interesting study was published by Nathiya et al. regarding the inhibitory effect of two expired drugs—Moxifoxacin and Betnesol—in 1 M H_2_SO_4_ solution at the same concentration of 400 ppm [154]. Under the same conditions, the *IE* for the first drug was approx. 95%, while for the second drug the *IE* was about 85%. The significant difference between *IE*s was due to the molecular structures. While Moxifoxacin contains in its molecule seven heteroatoms having lone pair electrons (four oxygen atoms and three nitrogen atoms), Betnesol contains only four nitrogen atoms; moreover, Moxifoxacin contains many more π electrons than Betnesol.

Concerns regarding identifying active expired drugs as inhibitors for aluminum alloys should also be noted. For AA5083 alloy, expired B1 vitamin demonstrated high *IE*s in 1 M HCl solution [160]; expired Thephylline for Al 7075 alloy in 1 M NaOH [157]; Phenylphrine for Al 2024 alloy in 1 M HCl solution [161].

In a couple of papers, expired drugs as inhibitors for nickel, tin and zinc have been presented (Table 9). Good results have been obtained for tin in 1 M HCl using expired drugs such as Primperan and E-mox [158], Novacid [162], Septazole and Pentoxifylline [163].

## 3. Drug Stability and Analysis

The issue of expired drug stability has to be solved, taking into account two aspects: stability in time under storage conditions and stability in the aggressive environments in which they are used as corrosion inhibitors. Unfortunately, many authors rely on the fact that, in expired drugs, the active substance remains unchanged for many years even after the expiration date. The 1985 study by the US Food and Drug Administration (carried out at the request of the Pentagon), showed that about 90% of the drugs in the US Army stock maintained their stability even after several years after the expiration date [28].

The analysis of drugs is a very topical problem that aims to evaluate the composition of pharmaceutical formulations, i.e., the structural integrity verification of both the active molecules and the excipients. A rigorous and systematic analysis guarantees, on the one hand, the favorable effects on human and animal health; on the other hand, it provides valuable information that can lead to the extension of the shelf life of some pharmaceutical products, which, following some initial studies, have been assigned a shorter period of time until expiration by competent institutions. Analysis of expired pharmaceutical forms is a complex process involving the use of physical, chemical and biological methods.

It is difficult to achieve an exhaustive classification of these methods. In principle, the physical and physicochemical methods include the control methods of the properties specified by the manufacturer, the qualitative separation and identification of the components, their quantitative dosing and the radioisotopic control. On the other hand, biological methods provide special information on the pharmacological action of active substances, which is why they are not useful in the study of drugs as corrosion inhibitors. All these methods are standardized and presented in reference works such as the *European Pharmacopoeia*, the US Food and Drug Administration guidelines and other works cited in the specialized literature. Drugs expired from an administrative point of view do not completely lose their pharmacological properties and their molecular structures do not degrade significantly. Sometimes, these structures are stable for a very long period of time and, even if they are unsuitable for administration to humans or animals, they can be used as corrosion inhibitors in various industries after confirming the integrity of their molecular structure through various analytical methods. This category of pharmaceutical substances used as corrosion inhibitors also includes various food supplements or natural extracts used in therapy.

It should be noted that most papers on expired drugs as corrosion inhibitors unfortunately do not contain references to the analysis of the active substance content. Even in cases where the authors have carried out such analyses, the results are not given either in the text of the paper or in the Supplementary Materials section.

In the research of expired drugs as corrosion inhibitors, Anaee et al. analyzed expired Metoclopramide by high-performance liquid chromatography, confirming its stability after the expiration date [167].

An interesting study demonstrating the integrity of the majority of the functional groups of Metoclopramide and Amoxicillin after the expiration date by means of Fourier-transform infrared spectroscopy (FT-IR) was carried out by Attia et al. [158]. They found that, in the case of expired Metoclopramide, the characteristic vibration band of the NH_2_ group disappeared. As well, the characteristic band of the amide group became wider and moved from 1200 cm^−1^ to 1600 cm^−1^. Moreover, in the case of Amoxicillin, the disappearance of the characteristic band of the NH_2_ group was found and the characteristic band of the OH group became very broad. Also, the disappearance of the bands’ characteristics of the C=O and C-S-C bond was observed. Compared with the IR spectrum of Amoxicillin within the expiration date, the appearance of two new bands characteristic of C-N and C=C bonds could be observed at wave numbers 1500 cm^−1^ and 1624 cm^−1^, respectively. This behavior could be due, first of all, to the presence of traces of water in commercial pharmaceutical preparations, which can initiate hydrolysis reactions of some functional groups, such as the NH_2_ group, with the formation of the OH group. On the other hand, structural transformations can also take place under the influence of oxygen in the air and/or sunlight, which can cause decarboxylation and heterocycle closing processes, as can be seen from the characteristic IR fingerprints [168,169].

Bustos-Terrones et al. [80] created a coating system for surfaces that have to be protected against corrosion using Fluconazole embedded in kaolinite. Attenuated total reflection (ATR) FT-IR investigations revealed that the molecular structure of Fluconazole was preserved. The characteristic absorption bands appeared at 1135 cm^−1^ for the C–F bond, the C–C bond of the aromatic ring was evident at 1520–1623 cm^−1^ and the characteristic bands of C–H and –OH bonds, respectively, appeared at the wave number 3100 cm^−1^. Similarly, through UV-Vis spectroscopy and reflection spectroscopy, Shamnamola et al. demonstrated the formation of a protective film of Cephapirin on steel surface, having a protective role against corrosion [170].

Hameed et al. demonstrated the stability of Voltaren by confirming the molecular integrity of the active substance by FT-IR [152]. The presence in the spectrum of a broad band at the wave number 3450 cm^−1^ confirmed the presence of the terminal hydroxyl group; the presence of a band at 810 cm^−1^ confirmed the integrity of the benzene nuclei in the structure of the molecule even if the pharmaceutical product had expired. Recently, the chemical stability of expired Tenoxicam on steel surfaces was investigated by Elabbasy and Gadow by FT-IR, confirming that the expired pharmaceutical product kept its structure unmodified on the surface on which it was adsorbed [88]. Similarly, Fouda et al. demonstrated that Clopidogrel could be used on steel surfaces in an aqueous environment as an ecological anticorrosion agent, keeping its molecular structure unchanged on the respective surface (a fact highlighted by FT-IR and UV-Vis spectrophotometric studies) [112].

Several authors have studied the electrochemical behavior of drugs in aggressive solutions by cyclic voltammetry. In this way, information was obtained on the stability of the drugs in a potential range closed to the corrosion potential of the studied metal. At the same time, indications were obtained on the adsorption capacity of the inhibitors, taking into account the displacement of or decrease in the peaks recorded in the base solution. Thus, Tasić, et al. studied the anodic behavior of expired Paracetamol in solutions simulating acid rain (0.2 M H_2_SO_4_) [141]. By tracing the cyclic voltammograms on the platinum electrode, the anodic potential range was widened and the inhibitors were subjected to more aggressive polarization. Under such conditions, the electrochemical behavior of Carbamazepine in 1 M H_2_SO_4_ solution was studied, which proved to be stable even under such circumstances [28].

A. Samide et al. used CV not only to study the electrochemical behavior of the inhibitor but also to identify the mechanism of the anticorrosive protection of copper in 1 M HCl solution using expired Metronidazole [145].

Additionally, some expired drugs still maintained their anticorrosive effectiveness even after decomposition by hydrolysis in the aggressive aqueous solutions because the hydrolysis products also had an inhibitory character. An example of such behavior was given by Streptomycin, which in acidic solutions hydrolyzed with the formation of Streptidine and Streptobiosamine, Streptose and Methyl-Glucosamine, all of which contained O and/or N heteroatoms with lone pair electrons [171].

## 4. Conclusions

Over the past decade, research on expired drugs as corrosion inhibitors has made significant progress. More than 100 expired drugs have been identified that exhibit corrosion inhibitory effects in aggressive solutions. Remarkable results have been obtained, especially for the corrosion inhibition of carbon steel and mild steel. High efficiencies have also been reported for copper and aluminum, as well as their alloys. Without any doubt, it can be stated that the research has concentrated mostly on commonly used metals and alloys. Test environments have been, in general, very aggressive dilute hydrochloric and sulfuric acid solutions used at an industrial level in pickling operations and descaling and cleaning of some machines. Tests in neutral solutions have been very little addressed, even if there were perspectives of using expired drugs as inhibitors in aqueous solutions from thermal cooling/heating plants (which have the advantage of being closed systems, thus avoiding environmental contamination).

The inhibitory efficiency of expired drugs has been determined by applying the same methods as for the case of fresh drugs. The methods mostly used were weight loss measurements, potentiodynamic polarization and electrochemical impedance spectroscopy. The following were used as complementary methods: volumetric method, thermometric measurements, atomic absorption spectroscopy, electrochemical frequency modulation and even acidimetric analysis. From Table 1, it can be seen that there were significant differences between the *IE*s obtained under the same conditions but using different methods. These differences appeared, first of all, due to the fact that some of the methods gave instantaneous values (potentiodynamic polarization, electrochemical impedance spectroscopy, electrochemical frequency modulation and thermometric measurements) and others gave results over a longer period of time (weight loss measurements, volumetric method, atomic absorption spectroscopy and acidimetric analysis). Secondly, differences also appeared due to the different accuracies of the measurements by the applied methods. It was expected that the precision of measurements for *IE*s would be limited to two, or at most three, significant figures. Presenting values with four or even five significant figures—as they appeared in several published articles—was an exaggeration or drafting error.

The stability of expired drugs is very important from the application point of view. Nevertheless, few researchers in the field have studied the active substance content of the researched medicines. The idea of analyzing expired drugs in order to determine the degree of degradation of the active substance, as well as the nature and toxicity of the degradation products, must be accepted. It is also important to evaluate the stability of expired drugs in the aggressive solutions in which they are used as inhibitors by cyclic voltammetry on an inert electrode after the prior determination of the open circuit potential.

Nevertheless, the enthusiasm of corrosion scientists regarding the use of expired drugs as corrosion inhibitors must be tempered for several reasons. First of all, not all expired drugs are “green inhibitors”. The high toxicity of some drugs proposed as inhibitors, such as some antibiotics, is very well known. On the other hand, the use of antibiotics as corrosion inhibitors increases the danger of inducing resistance to microorganisms, with repercussions on human and animal health that are difficult to estimate.

To ensure that research results from the study of expired drugs are applied in industrial practice and not just at the academic level, a number of actions have been identified as being of utmost importance [25]:

- Identification of new expired medicines that are really friendly to the environment in aggressive solutions, including neutral and basic ones, which are preferably used in closed systems to avoid contact with the environment;
- Analysis of the effectiveness of expired drugs in relation with that of fresh drugs;
- Evaluation of the active substance content of expired drugs and the stability in aggressive solutions;
- Study of the influence of excipients from expired drugs on the inhibitory efficiency;
- Stimulation of research to develop new procedures of selective collection and delivery of expired medicines in enough quantities for the proposed purpose;
- Study of the legislative framework that allows the use of expired drugs as corrosion inhibitors;
- Development of disposal methods for exhausted solutions containing expired medicines;
- Collaboration of corrosion engineers with pharmaceutic experts.

## Figures and Tables

**Table 1:** Inhibitory efficiency of expired drugs for mild steel in different corrosive environments.

Corrosive Solution	Expired Drug	Inhibitor Concentration	IE (%) (Experimental Method)	Reference
1 M HCl	Ambroxol	9%	64.89 (WL); 60.52 (PDP); 61.95 (EIS);	[45]
	Amoxicillin	1800 ppm	94.47 (WL)	[46]
	Asthaline	0.9% *v*/*v*	62.35 (WL); 55.83 (PDP); 55.04 (EIS)	[47]
	Atenolol	200 ppm	91.04 (PDP); 93.29 (EIS)	[48]
	Atorvastatin (expired)	150 ppm	99.08 (PDP); 96.38 (EIS); 96.47 (WL)	[49]
	Atorvastatin (fresh)	150 ppm	98.41 (PDP); 96.36 (EIS); 93.52 (WL)	[49]
	Cefdinir	6.32 × 10^−4^ M	96.9 (EIS); 95.6 (PDP)	[50]
	Chlorpromazine	700 ppm	85.1 (PDP)	[51]
	Declophen	2.5% *v*/*v*	86.6 (WL); 87.5 (PDP)	[52]
	Doxofylline	200 ppm	72.8 (WL); 72.1 (PDP); 72.6 (EIS)	[53]
	Dulcolax	500 ppm	91.8 (WL); 91.0 (VOL); 92.5 (PDP); 93.0 (EIS)	[54]
	Gentamicin	0.9% *v*/*v*	76.65 (WL); 75.06 (PDP); 78.44 (EIS)	[55]
	Helicure	300 ppm	83.2 (WL); 84.6 (EFM); 83.8 (EIS); 85.8 (PDP)	[56]
	Irnocam	600 ppm	80.4 (WL); 75.4 (PDP)	[57]
	Lumerax	100 mg/L	98.5 (WL); 97.6 (EIS); 95.2 (PDP)	[58]
	Lupicof	0.9%	79.2 (WL); 58.24 (PDP); 70.86 (EIS)	[59]
	Mebeverine	700 ppm	82.1 (PDP)	[51]
	Meloxicam	30 ppm	90.2 (WL); 79.2 (EFM); 80.8 (EIS); 88.0 (PDP)	[60]
	Metoclopramide	700 ppm	83.3 (PDP)	[51]
	Metronidazole	10^−5^ M	62.14 (WL); 72.82 (PDP); 64.62 (EIS)	[61]
	Nifedipine	200 ppm	93.13 (PDP); 95.61 (EIS)	[48]
	Ornidazole	2·10^−5^ M	71.10 (WL); 79.04 (PDP); 71.61 (EIS)	[61]
	Ranitidine	400 ppm	89 (WL); 90 (PDP); 92 (EIS)	[62]
	Tinidazole	10^−5^ M	83.53 (WL); 85.21 (PDP); 80.92 (EIS)	[61]
	Tramadol	100 ppm	96.12 (WL); 97.2 (EIS)	[63]
5% HCl	Ceftin	0.4 mg/L	83.308 (WL); 94.401 (AAS)	[64]
0.5 M HCl	Ethambutol	1000 ppm	99.60 (WL); 97.61 (PDP); 93.72 (EIS)	[65]
2 M HCl	Acetazolamide	500 ppm	82.4 (WL); 93.3 (PDP); 96.4 (EIS)	[66]
	Bactrim	400 ppm	67.9 (WL); 94.4 (PDP); 93.1 (EIS)	[67]
3 M HCl	Abacavir	0.4 g/L	85.454 (WL); 95.365 (PDP); 72.648 (EIS)	[68]
	Amitriptyline	2 mg/L	87.577 (PDP); 82.3 (EIS)	[69]
	Doxercalciferol	0.4 mg/L	95.4 (AAS); 86.967 (PDP); 88.617 (EIS)	[70]
	Fluoxymesterone	0.2 mg/L	94.200 (AAS); 87.517 (PDP); 88.813 (EIS)	[71]
	Lorazepam	0.4 mg/L	82.72 (WL); 96.49 (AAS); 87.70 (PDP)	[72]
0.5 M H_2_SO_4_	Glibenclamide	500 ppm	86.3 (WL); 85.1 (PDP); 86.5 (ESI)	[73]
	Glimepiride	500 ppm	86.3 (WL); 88.0 (PDP); 90.4 (EIS)	[73]
	Tramadol	500 ppm	93.2 (WL); 93.6 (VOL); 94.0 (ACI)	[44]
1 M H_2_SO_4_	Ambroxol	9% *v*/*v*	81.10 (WL); 68.61 (PDP); 68.93 (EIS)	[74]
	Ampicillin	400 ppm	93.17 (WL); 93.95 (PDP); 91.02 (EIS)	[75]
	Asthalin	9% *v*/*v*	73.68 (WL); 75.56 (PDP); 69.23 (EIS)	[76]
	Azithromycin	400 ppm	88.32 (PDP); 87.20 (EIS); 84.97 (WL)	[77]
	Chlorpromazine	700 ppm	97.13 (PDP)	[51]
	Flucloxacillin	400 ppm	88.20 (WL); 90.92 (PDP); 88.99 (EIS)	[75]
	Formoterol	300 ppm	95 (WL); 98 (EIS); 95 (PDP)	[78]
	Mebeverine	700 ppm	93.62 (PDP)	[51]
	Metoclopramide	700 ppm	96.42 (PDP)	[51]
	Roxithromycin	400 ppm	84.91 (PDP); 86.75 (EIS); 86.12 (WL)	[77]
1 M HNO_3_	Kinurenic acid	300 ppm	88.9 (WL); 89.8 (PDP); 89.3 (EIS)	[79]
0.1 M Na_2_SO_4_ + 3% NaCl	Fluconazole	150 ppm	84 (PDP); 80 (EIS)	[80]
	Lansoprazol	100; 50 ppm	95 (PDP); 75 (EIS)	
3.5% NaCl	Rabeprazole Domperidone Benfotiamine	0.5 mg/L	98.52 (WL); 98.81 (WL); 98.92 (WL)	[81]
NaCl 700 ppm	Acarbose	100 ppm	82.36 (WL); 75.80 (EIS); 86.22 (PDP)	[82]
	Voglibose		79.73 (WL); 74.75 (EIS); 83.97 (PDP)	
	Miglitol		78.25 (WL); 70.25 (EEIS); 81.56 (PDP)	
0.625 M NaCl	Artemether-Lumefantrine		75	[83]
2.6% NaCl + 0.4% (NH_4_)_2_SO_4_	Fluconazole	150 ppm	84.46 (PDP); 64.83 (EIS)	[84]
Oilfield fluid	Salbutamol	0.4 g L^−1^	80.1 (WL); 89.0 (PDP); 84.8 (EIS)	[85]

**Table 2:** Inhibitory efficiency of expired drugs for carbon steel in different corrosive environments.

Corrosive Solution	Expired Drug	Inhibitor Conc.	IE (%) (Experimental Method)	Reference
0.5 M HCl	Amiloride	7 × 10^−3^ M	99.2 (TM); 98.6 (WL); 92.8 (EIS); 98.12 (PDP)	[87]
	Tenoxicam	4 × 10^−4^ M	81.0 (PDP); 71.0 (EIS)	[88]
1 M HCl	Amlodipine Besylate	250 ppm	84.0 (WL); 83.5 (PDP); 94.3 (EIS)	[89]
	Amlodipine besylate	250 ppm	84.0 (WL); 83.5 (PDP); 94.3 (EIS)	[90]
	Aripiprazole	300 ppm	93.1 (WL); 94.8 (PDP); 95.1 (EIS)	[91]
	Augmentine	300 ppm	97.8 (WL); 97.1 (PDP); 98.0 (ACI)	[92]
	Bromocriptine	1.12 × 10^−4^ M	82.6 (WL); 88.8 (PDP); 85.1 (EIS)	[93]
	Bupropion	500 ppm	90.3 (PDP); 88.0 (EIS)	[94]
	Carbocysteine	2.4%	61 (WL)	[95]
	Carvedilol	1.6 × 10^−4^ M	98.9 (WL); 98.1 (PDP); 98.6 (EIS); 98.1 (EFM)	[96]
	Desloratidine	1.93 × 10^−4^	85.2 (PDP); 92.7 (WL)	[97]
	Farcolin	20% *v*/*v*	95 (PDP); 97 (WL)	[98]
	Immulant	1.2 g L^−1^	97.5 (PDP); 95.3 (EIS)	[99]
	Indomethacin	500 ppm	83.81 (WL); 79.61 (PDP); 82.37 (EIS)	[100]
	Megavit	300 ppm	91.7 (PDP); 90.6 (EIS)	[101]
	Metronidazole	0.8 mmol L^−1^	80.6 (PDP); 81.1 (EIS); 80.0 (WL)	[102]
	Nitazoxanide	300 ppm	90.1 (WL); 90.2 (EIS); 90.0 (PDP)	[103]
	No Plac	20 ppm	93.0 (WL); 77.8 (PDP); 91.6 (EIS)	[104]
	Omeprazole	0.2 × 10^−3^ M	96.6; 98.7; 98.0	[105]
	Omeprazole	40 mg L^−1^	70 (PDP); 90 (WL); 92 (EIS)	[106]
	Pantoprazole sodium	300 ppm	93.3 (WL); 95.1 (PDP); 92.2 (EIS); 89.2 (EFM)	[107]
	Paracetamol	2.4%	68 (WL)	[95]
	Paracetamol	10^−3^ M	85.8 (PDP); 84.8 (EIS)	[108]
	Phenytoin	500 ppm	79.1 (WL); 81.78 (PDP); 79.0 (EIS)	[109]
	Podocip	100 mg/L	96.57 (WL); 97.93 (EIS); 97.55 (PDP)	[110]
2 M HCl	Captopril	15 × 10^−5^ M	77.9 (WL); 65.4 (PDP); 81.7 (EIS)	[111]
	Clopidogrel	250 ppm	82.8 (WL); 93.2 (PDP); 85.8 (EIS); 86.4 (EFM)	[112]
	Guaifenesin	15 × 10^−5^ M	71.4 (WL); 61.2 (PDP); 80.3 (EIS)	[111]
	Hydrochlorothiazide	15 × 10^−5^ M	81.8 (WL); 73.5 (PDP); 83.1 (EIS)	[111]
	Tobramycin	500 ppm	90.5 (PDP); 84.3 (EIS); 80.0 ™	[113]
15% HCl	Metformin	1000 ppm	68.79 (WL); 74.72 (EIS); 73.46 (PDP)	[114]
0.1 M H_2_SO_4_	Cefotaxime	10^−3^ M	78.2 (EIS); 79.2 (PDP); 74.7 (WL)	[115]
	Ceftriaxone	10^−3^ M	84.4 (EIS); 85.0 (PDP); 80.8 (WL)	[115]
	Cefuroxime	10^−3^ M	71.5 (EIS); 71.8 (PDP); 68.5 (WL)	[115]
0.5 M H_2_SO_4_	Amiloride	7 × 10^−3^ M	87.3(TM); 88.2 (WL); 90.8 (EIS); 89.5 (PDP)	[87]
	Bupropion	500 ppm	82 (PDP); 83 (EIS)	[94]
	Omeprazole	40 mg L^−1^	70 (PDP); 90 (WL); 92 (EIS)	[106]
	Paracetamol	10^−3^ M	93.7 (PDP); 96.4 (EIS)	[108]
1 M H_2_SO_4_	Neomycin		79.5 (PDP); 73.2 (EIS)	[116]
	Paracetamol	275 mg/L	94.2 (PDP); 92.1 (EIS)	[117]
0.5 M H_3_PO_4_	Etoricoxib	225 ppm	80.63 (PDP)	[118]
1 M H_3_PO_4_	Esomeprazole	10^−4^ mol L^−1^	99.55 (PDP); 99.52 (EIS)	[119]
3 M H_3_PO_4_	Lansoprazole	10 mmol L^−1^	92.9 (PDP); 93.5 (EIS)	[120]
	Raboprazole	10 mmol L^−1^	94.8 (PDP); 94.2 (EIS)	[120]
3% NaCl	Alprazolam	0.4 g/L	85.78 (WL); 80.00 (AAS)	[121]
	Perindopril	0.4 g/L	90.00 (WL); 91.58 (AAS)	[121]
3.5% NaCl	Zosyn	10^−3^ M	91.08 (PDP)	[122]
5% NaCl	Oxazepam	0.4 mg/L	80.113 (WL); 80.114 (VOL)	[123]
2 M NaCl	Paracetamol+Zn^+2^	200 ppm + 100 ppm	91.0 (PDP); 92.1 (EIS); 92.0 (TM); 94.4 (WL); 83.8 (AAS)	[124]
0.5 M CH_3_COOH- 0.25 M CH_3_COONa	Streptomycin	10^−3^M	82.1 (WL); 87.4 (PDP); 82.2 (EIS)	[125]
0.5 M NaHCO_3_	Amiloride	7 × 10^−3^ M	96.6 (TM); 92.2 (WL); 91.9 (EIS); 93.5 (PDP)	[87]
H_2_SO_4_ 0.1 M/CH_3_COOH 0.25 M/CH_3_COONa 0.25 M	Carbamazepin	5 × 10^−3^ M	90 (PDP)	[28]
	Paracetamol	5 × 10^−3^ M	85 (PDP)	[28]
CO_2_ sat. 3.5% NaCl + 340 ppm acetic acid	Metformin	200 ppm	89.47 ± 1.42 (EIS); 86.31 (PDP)	[126]

**Table 3:** Inhibitory efficiency of expired drugs for SABIC Fe in different corrosive environments.

Corrosive Solution	Expired Drug	Inhibitor Conc.	IE (%) (Experimental Method)	Reference
1 M HCl	Acyclovir	500 ppm	87.0 (WL); 94.47 (PDP); 93.82 (EIS)	[127]
	Amoxicillin		87.0 (WL); 94.47 (PDP); 93.82 (EIS)	[129]
	Ampicillin		91.8 (WL); 95.79 (PDP); 95.83 (EIS)	[127]
	Ceftriaxone		96.9 (WL); 98.02 (PDP); 97.90 (EIS)	
	Cefuroxime		92.9 (WL); 96.58 (PDP); 96.00 (EIS)	[129]
	Omeprazole		88.8 (WL); 95.26 (PDP); 95.50 (EIS)	[128]
0.5 M H_2_SO_4_	Vitamin B1	250 mg L^−1^	89.45 (WL); 91.14 (PDP); 89.12 (EIS)	[130]
	Vitamin B2		91.49 (WL); 92.40 (PDP); 90.85 (EIS)	

**Table 4:** Inhibitory efficiency of expired drugs for cast iron in different corrosive environments.

Corrosive Solution	Expired Drug	Inhibitor Conc.	IE (%) (Experimental Method)	Reference
1 M HCl	Cefpodoxime	240 ppm	95.2 (WL); 94.4 (PDP); 93.4 (EIS)	[131]
	Levofloxacin		93.4 (WL); 92.4 (PDP); 92.6 (EIS)	
	Linezolid		89.8 (WL); 89.5 (PDP); 87.7 (EIS)	
	Ofloxacin		91.6 (WL); 91.4 (PDP); 90.6 (EIS)	

**Table 5:** Inhibitory efficiency of expired drugs for pipeline steel in different corrosive environments.

Corrosive Solution	Expired Drug	Inhibitor Conc.	IE (%) (Experimental Method)	Reference
1 M HCl	Desloratidine	1.93·10^−4^ M	92.7 (WL); 85.2 (PDP)	[97]
1 M HCl	Tamsulosin	2·10^−3^ M	88.0 (EIS); 94.1 (PDP)	[134]
15% HCl	Pregabalin	2.5 g/L	91.5 (PDP); 92.4 (EIS); 96.5 (WL)	[132]
1 M H_2_SO_4_	Co-amoxiclav	300 ppm	96 (WL); 95.3 (VOL); 95 (ACI); 96 (TM)	[133]

**Table 6:** Inhibitory efficiency of expired drugs for stainless steel AISI 304.

Corrosive Solution	Expired Drug	Inhibitor Conc.	IE (%) (Experimental Method)	Reference
1 M HCl	Metronidazole	0.8 mmol L^−1^	67.9 (WL) 67.9 (PDP); 68.3 (EIS);	[102]
2 M HCl	Concor	300 ppm	75.10 (WL); 64.1 (PDP); 85.8 (EIS); 81.2 (EFM)	[136]
0.5 M H_2_SO_4_	Levothyroxine	150 ppm	94.3 (WL); 93.2 (PDP); 91.7 (EIS)	[135]
3.5% NaCl	Cefalexim	-	-	[137]

**Table 8:** Inhibitory efficiency of expired drugs for aluminum and aluminum alloys.

Sample	Corrosive Solution	Expired Drug	Inhibitor Conc.	IE (%) (Experimental Method)	Reference
Al	1 M HCl	Cidamex	300 ppm	90.9 (WL); 98.7 (VOL); 99.6 (PDP); 81.0 (EIS); 84.3 (EFM)	[153]
	1 M HCl	Metronidazole	0.8 mmol L^−1^	88.3 (WL); 88.2 (PDP); 91.1 (EIS);	[102]
	1 M HCl	Micardis	300 mg L^−1^	96.8 (WL); 97.2 (PDP); 94.0 (EIS); 99.4 (VOL)	[155]
	1 M HCl	Ranitidine	300 ppm	82 (WL); 82.2 (PDP)	[27]
	1 M HCl	Voltaren	125 ppm	89.7 (WL); 91.7 (PDP)	[152]
	3 M HCl	Oseltamivir	2 mg/L	91.3 (WL); 98.639 (PDP); 98.8 (AAS)	[150]
	3 M HCl	Naproxen	1 g/L	99.002 (PDP); 97.704 (EIS); 97.2 (VOL)	[151]
	3 M HCl	Atenolol	0.4 g/L	87.868 (WL); 98.666 (PDP); 95.878 (EIS); 97.000 (AAS); 92.063 (VOL)	[149]
	1 M H_2_SO_4_	Moxifoxacin	400 ppm	95.31 (WL); 95.40 (PDP); 94.00 (EIS)	[154]
		Betnesol		86.17 (WL); 85.01 (PDP); 85.47 (EIS)	
	1 M NaCl	Linezolid	250 mg L^−1^	77.2 (WL); 85.8 (PDP); 83.7 (EIS)	[156]
		Norfloxacin		74.2 (WL); 79.5 (PDP); 77.2 (EIS)	
Al alloy 7075	1 M NaOH	Theophylline	2.5%	90 (EIS); 91 (PDP)	[157]
Al alloy 2024	1 M HCl	Phenylphrine	500 ppm	82.96 (WL)	[158]
Al alloy 6061	0.1 M HCl	Diclofenac	150 ppm	99.98 (PDP)	[159]
Al alloy 6061	3.5% NaCl	Diclofenac	150 ppm	85.98 (PDP)	

**Table 9:** Inhibitory efficiency of expired drugs for nickel, tin and zinc.

Metal	Corrosive Solution	EXPIRED DRUG	Inhibitor Conc.	IE (%) (Experimental Method)	Reference
Ni	1 M HCl	Ceftriaxone	10^−5^ M	71.4 (WL); 73.0 (PDP); 73.9 (EIS)	[164]
	0.5 M H_2_SO_4_	Ceftriaxone	10^−4^ M	69.6 (WL); 70.1 (PDP); 69.5 (EIS)	
Sn	1 M HCl	Primperan	9.9% *v*/*v*	96.45 (PDP)	[158]
		E-mox		92.05 (PDP)	
		Primperan		91.32 (WL)	[165]
		E-mox		93.85 (WL)	
		Novacid		93.9 (WL); 94.2 (PDP)	[162]
		Septazole	9% *v*/*v*	95 (PDP)	[166]
		Septrin		90 (PDP)	
Zn	0.1 M HCl	Pentoxifylline	300 ppm	84.3 (WL); 71.2 (PDP); 83.6 (EIS)	[163]

## Data Availability

All data contained within the article.
